# Examining the effect of salbutamol use in ozone air pollution by people with exercise‐induced bronchoconstriction

**DOI:** 10.14814/phy2.70117

**Published:** 2024-10-29

**Authors:** Bennett T. Stothers, Andy Hung, Patric E. O. Gonçalves, Lulu X. Pei, Tessa van de Kerkhof, Jem I. Arnold, Owen D. Harris, Nadine Borduas‐Dedekind, Andrew W. Sheel, Michael S. Koehle

**Affiliations:** ^1^ University of British Columbia Vancouver Canada; ^2^ Radboud Universiteit Nijmegen The Netherlands

**Keywords:** Airway, asthma, exercise

## Abstract

Previous studies based on animal models have raised concerns about salbutamol use in ozone air pollution with regard to ozone related lung injury. We conducted a double‐blind, randomized, placebo‐controlled crossover study including 18 subjects diagnosed with EIB by a eucapnic voluntary hyperpnea (EVH) test. Participants completed 30 min of standardized moderate to vigorous exercise in four conditions: ozone plus salbutamol; room air plus salbutamol; ozone plus placebo medication; and room air plus placebo medication. Spirometry, fraction of exhaled nitric oxide, and symptoms were measured before, immediately after, 30 min after and 1 h after exercise. Measurements between the four conditions were compared using percent change from pre to post exercise. There was a statistically significant difference between the salbutamol and placebo medication groups for spirometric variables including FEV1 (Estimate = 6.3, 95% CI: 4.23–8.37, *p* < 0.001). No differences were observed between ozone and room air exposures. There were no significant differences in FeNO response between experimental conditions. We found that salbutamol improved pulmonary function in individuals with EIB when exercising in ozone and did not increase eosinophilic airway inflammation as indicated by FeNO. This evidence suggests that it is safe for people with EIB to continue to use salbutamol as proscribed when ozone levels are elevated.

## 1 INTRODUCTION

Physical activity is a well‐recognized component of health. Increased levels of daily physical activity can prevent numerous chronic health conditions such as cardiovascular disease, type two diabetes, and dementia (Fiuza‐Luces et al., 2018; Gill & Cooper, 2008; Middleton & Yaffe, 2009). Lower levels of regular physical activity has been linked to a 20%–30% increase in mortality (Fletcher et al., 2018). Many forms of physical activity take place outdoors, where people can be exposed to ambient air pollution, including particulate matter and ozone, which is a major global health concern linked to 4.2 million pre‐mature deaths each year (World Health Organization, n.d.). Physical activity‐induced increases in ventilation augment the inhaled dose of air pollution, raising concerns about the safety of exercising in poor air quality (Giles & Koehle, 2014). Although chronic exposures to air pollution have been related to poor health outcomes, evidence is mixed as to whether the risks associated with acute exposures outweigh the long term benefits of exercise (Hung et al., 2022). Evidence is further limited for individuals with pre‐existing conditions (e.g., asthma), who are considered particularly susceptible to air pollution toxicity (Guarnieri & Balmes, 2014).

Ground‐level ozone is a pollutant produced in photochemical smog when volatile organic compounds and NOx gases react in the presence of UV light (Jacob, 2000). Ozone exposure has been related to both increased frequency of asthma and exercise‐induced bronchoconstriction (EIB) exacerbations, and increased asthma prevalence (Rundell et al., 2015). Experimentally, in people with asthma, acute exposures during intermittent exercise can impair forced vital capacity (FVC), forced expiratory volume in 1 s (FEV_1_), and specific airway resistance (SRaw) (Scannell et al., 1996). With climate change, the frequency and severity of heat events are increasing, leading to higher levels of ozone exposure for people with asthma and/or EIB (Horton et al., 2016).

Salbutamol is a short acting beta‐2‐agonist commonly used by people with asthma and EIB before exercise to limit respiratory symptoms (Moffatt et al., 2022). However, with regard to exercise and air pollution, concerns have been raised that bronchodilation induced by salbutamol could allow pollutants to reach further down the airway causing greater potential for local effects and translocation to the systemic circulation. A previous study showed there were no differences in lung function between the filtered air and diesel exhaust in both salbutamol and placebo conditions (Koch et al., 2021). However, this relationship has not been investigated in ozone pollution and previous animal studies have demonstrated the bronchodilators may exacerbate ozone related lung inflammation (Henriquez et al., 2019; Henriquez, Snow, Schladweiler, Miller, Dye, Ledbetter, Richards, Hargrove, et al., 2018; Henriquez, Snow, Schladweiler, Miller, Dye, Ledbetter, Richards, Mauge‐Lewis, et al., 2018).

The effect of salbutamol on lung function and inflammation needs to be quantified in people with EIB to inform on exercising recommendations during air pollution events. Therefore, this study had two main objectives. First, to determine the effect that salbutamol has on lung function in people with EIB performing exercise when exposed to ozone air pollution. Second, to quantify the effect that salbutamol has on lung inflammation. We hypothesized that salbutamol use prior to exercise would improve post‐exercise lung function but also increase lung inflammation in people with EIB (Horton et al., 2016).

## 2 METHODS

### 2.1 Participants and recruitment

Inclusion criteria were: EIB (confirmed by a eucapnic voluntary hyperpnea (EVH) test), ability to safely perform maximal‐intensity exercise, and age 18–50 years. Exclusion criteria included: use of inhaled corticosteroids, history of smoking, presence of chronic respiratory diseases other than asthma/EIB, upper respiratory tract infection within the last 4 months, pregnancy, or allergy to salbutamol. Neither the public or research participants were involved in the design, conduct, reporting, or dissemination plans of our research.

### 2.2 Experimental design and variables

This study utilized a placebo‐controlled, double‐blinded, crossover design. Eligible participants exercised for 30 min in each combination of air quality and medication: ozone + salbutamol, ozone + placebo, room air + salbutamol, room air + placebo. A Latin square design was used, with four orders of exposure that were uniform both within periods and within sequences (Saville & Wood, 1991). Ozone concentrations was standardized (ACT‐5000, Mellifiq, Hägersten, Sweden) to 170 ppb, the upper range of realistic concentrations as a 1‐h average (Lu et al., 2018). This mixing ratio was chosen to represent the upper end of a high‐level ozone pollution. A sampling port for a calibrated 49iQ Ozone Analyzer (Thermo Scientific, Waltham, MA, USA) on the inspired side of the participant monitored inhaled ozone dose. Participants rode a Velotron cycle ergometer (Racemate Inc., Seattle, WA, USA) while wearing nose clips and breathing from a two‐way, non‐rebreathing mouthpiece (Hans Rudolf, Kansas, USA). The medication condition comprised either 200 μg salbutamol or placebo. Participants were provided standardized instruction to take 2 × 100 μg inhaled salbutamol or placebo inhalant, from an inhaler training device with no active medication, using a meter‐dose inhaler (MDI) (Sanchis et al., 2013) with a spacer (Newman, 2004).

Spirometry was conducted in accordance with American Thoracic Society Guidelines (Graham et al., 2019) and measured using a metabolic cart (TrueOne 2400, Parvo Medics Inc., UT, USA). The fraction of exhaled nitric oxide (FeNO) was measured in quadruplicate at each time point using an NObreath‐v2 (Bedfont Scientific Ltd., Kent, England); the first measurement was disregarded and the last three were averaged. Symptoms measured included dyspnea, cough, sore throat, headache, chest pain, and chest tightness. Dyspnea was rated on a scale of 0–10 and all other symptoms were measured on a scale of 0–5.

### 2.3 Visits and procedures

**FIGURE 1:**
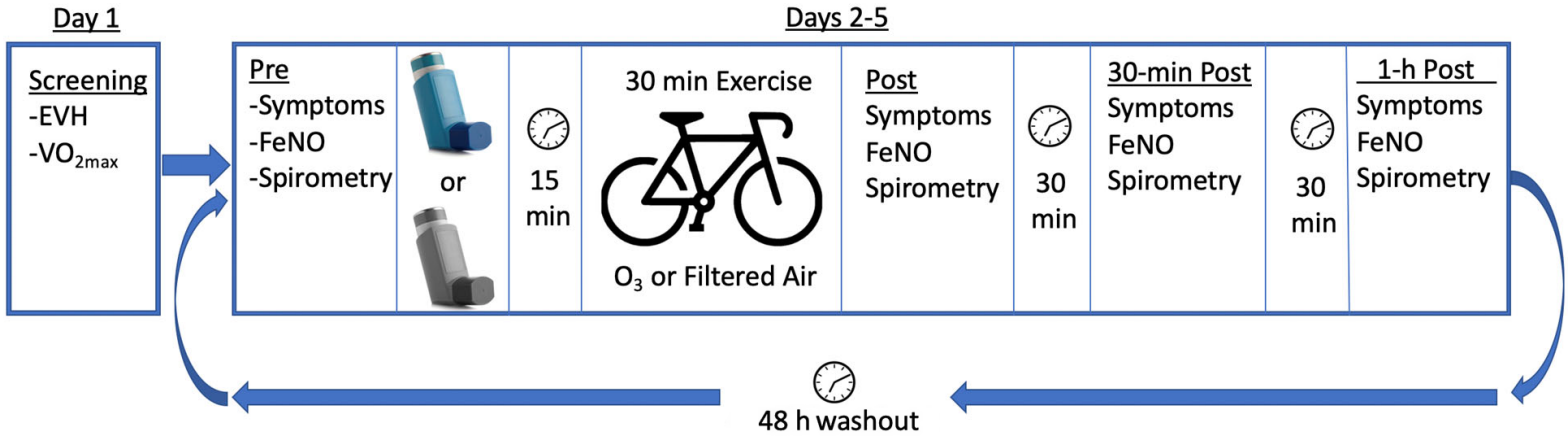
Summary of Study Protocol.

This study involved five visits to the lab and is summarized in Figure 1.

Before each visit, participants were asked to avoid anti‐histamines for 72 h, long‐acting beta‐agonists for 48 h, leukotriene modifiers for 24 h, and short‐acting beta agonists for 8 h before each visit. Participants were asked to avoid foods high in nitrates for 12 h before each visit and asked to avoid vitamin C and E supplements for the duration of the study. Participants were also asked to avoid exercise 24 h before each visit. Day one served as a screening day, during which an EVH test was administered to confirm the presence of EIB (Anderson et al., 2001). A fall index (i.e., the percent drop in FEV_1_ from pre‐ to post‐test) of 10% or greater was considered a positive test (Anderson et al., 2001). Eligible participants then took 200 μg salbutamol and waited 15 min before starting a VO_2max_ test. The VO_2max_ test followed a ramp protocol and expired gas was analyzed using the TrueOne metabolic cart. Following a self‐selected warm‐up, participants started at 30 W, with the workload increasing by 1 W every 3 s for women and every 2 s for men until (1) they could no longer continue, or (2) they could no longer maintain a cadence of 60 rpm.

The next four lab visits constituted the experimental trials. Pre‐exercise FeNO, symptoms, and spirometry were collected, and then participants inhaled two puffs of either placebo or salbutamol. After 15 min, participants cycled at 60% of peak wattage for 30 min to represent realistic moderate‐to‐intense exercise. Dependent variables were again collected immediately, 30‐min, and 1‐h post‐exercise. All visits were separated by 48 h to minimize carryover effects from previous exposure o ozone or salbutamol.

### 2.4 Statistical analysis

Baseline characteristics were summarized descriptively for the whole sample as well as disaggregated by sex. Mean ozone concentrations were compared between conditions using a one‐way repeated measures ANOVA with subsequent post hoc *t*‐tests to determine potential differences in air quality between the air quality conditions.

Each response variable was analyzed independently using a linear mixed effect model to estimate the change compared to baseline after the exercise intervention under each condition. For FEV_1_/FVC and FeNO, the change in absolute units from baseline was analyzed. FEV_1_, FVC, and FEF_25‐75_ were analyzed as a relative percent change from baseline.

Fixed effects were *medication* (placebo vs. salbutamol) and *air quality* (8–9 ppb in room air vs. 170 ppb of ozone). An initial model also included a fixed effect of sample time as a categorical factor across three post‐exercise time periods (immediate, 30‐min, and 60‐min post). However, post hoc testing revealed no differences between any sample periods for any response variables. Therefore, a mean score was calculated for each participant across the three sample times in each condition, and a reduced model was compared with Akaike's Information Criterion (AIC) and Bayesian Information Criterion (BIC) (Van der Elst et al., 2016). The reduced model was selected for all response variables based on lower AIC and BIC scores. All subsequent model results are reported from this.

Random effects of participant were included for the intercept of the models. Pairwise post hoc tests of estimated marginal contrasts were performed using the *emmeans* R package for each level of the fixed effects (Lenth et al., 2023). Degrees of freedom were estimated using Kenward‐Roger method and multiple comparisons were adjusted with the Tukey method for comparing families of 4 paired estimates. Alpha was set at *p* < 0.05.

Symptom severity scores were summarized across experimental conditions using medians (IQR) because of the skewed severity distributions. Side‐by‐side boxplots were used to visualize symptom severity at the different time points. Statistical analyses were conducted in Excel and R version 4.3.1 (R Core Team 2021).

## 3 RESULTS

### 3.1 Participant and exercise condition descriptions

In total, 36 people were screened, with 23 presenting with a positive EVH test, and 18 finishing all the required study visits. Five dropped out due to inability to schedule or finish all required visits. The baseline characteristics of the 18 completed participants are summarized in Table 1.

**TABLE 1:** Baseline characteristics of participants.

	All (*n* = 18)	Male (*n* = 8)	Female (*n* = 10)
Age (years)^a^	25.5 (5.2)	25.4 (3.3)	25.6 (6.4)
Height (cm)^a^	171.6 (8.6)	177.4 (5.2)	166.9 (7.9)
Weight (kg)^a^	71.1 (12.6)	79.6 (11.9)	64.3 (8.6)
VO_2peak_ (mL/kg/min)^a^	39.3 (8.4)	41.7 (7.3)	37.7 (8.8)
Fall index (%)^a^	18.2 (11.2)	16.6 (7.0)	19.4 (13.8)
Previous Asthma diagnosis^a^	15 (83.3%)	6 (75%)	9 (90%)
FeNO (ppb)^a^, ^b^	21.2 (21.5)	22.1 (23.3)	20.6 (20.3)
Spirometry^a^, ^b^			
FVC (L)	4.7 (1.0)	5.5 (0.7)	4.1 (0.7)
FEV_1_ (L)	3.6 (0.7)	4.1 (0.4)	3.2 (0.6)
FEV_1_/FVC	0.76 (0.05)	0.75 (0.07)	0.77 (0.04)
FEF_25‐75_ (L/min)	3.4 (1.0)	3.5 (0.8)	2.7 (0.7)

^a^ Values are mean (SD) or *n* (%).

^b^ Values are averages of all pre‐exercise measurements (four per participant).

Ozone levels are shown in Table 2, with no significant differences between ozone conditions (*p* = 0.94) and room air conditions (*p* = 0.48). The laboratory was climate‐controlled at 23°C and 40% relative humidity. No difference was found between baseline FeNO and spirometry values for each condition.

**TABLE 2:** Ozone levels in each of the conditions.

	O_3_ + PLA	O_3_ + SAL	RA + PLA	RA + SAL	*p*‐value
Ozone (ppb)^a^	171.9 (6.2)	172.1 (9.1)	8.3 (6.6)	9.2 (7.3)	< 0.001***

Abbreviations: O_3_, Ozone; PLA, Placebo medication; RA, Room Air; SAL, 200 μg of salbutamol.

^a^ Values are mean (SD).

*** Indicated *p* < 0.001 for ozone levels in O3 conditions versus ozone levels in RA conditions.

The estimates from the linear mixed effects models for each fixed effect with confidence intervals and *p*‐values are summarized in Table 3. The mean percent change in FVC, FEV_1_, and FEF_25‐75_ and the absolute change in FEV_1_/FVC and FeNO are reported in Table 4.

**TABLE 3:** Results of mixed effects models.

	Fixed effects	Estimate	95% CI (low, high)	*p*‐value
% Change in FVC	Intercept	−1.44	−2.35, −0.54	0.002**
	Salbutamol	1.83	0.67, 3.00	0.003**
	Ozone	0.58	−1.09, 1.23	0.907
	Salbutamol × Ozone	0.31	−1.33, 1.95	0.707
	*R* ^2^ Marginal	0.22		
	*R* ^2^ Conditional	0.36		
% Change in FEV_1_	Intercept	−0.35	−2.08, 1.39	0.69
	Salbutamol	6.30	4.23, 8.37	<0.001***
	Ozone	−1.04	−3.11, 1.03	0.32
	Salbutamol × Ozone	2.06	−0.87, 4.99	0.16
	*R* ^2^ Marginal	0.51		
	*R* ^2^ Conditional	0.65		
Change in FEV_1_/FVC	Intercept	0.82	−0.40, 2.04	0.18
	Salbutamol	3.40	2.07, 4.74	<0.001***
	Ozone	−0.87	−2.21, 0.46	1.20
	Salbutamol × Ozone	1.20	−0.68, 3.09	0.21
	*R* ^2^ Marginal	0.39		
	*R* ^2^ Conditional	0.63		
% Change in FEF_25‐75_	Intercept	3.88	−1.81, 9.58	0.18
	Salbutamol	16.30	9.33, 23.40	<0.001***
	Ozone	−4.89	−11.90, 2.13	0.17
	Salbutamol × Ozone	7.02	−2.90, 16.90	0.16
	*R* ^2^ Marginal	0.42		
	*R* ^2^ Conditional	0.56		
Change in FeNO (ppb)	Intercept	2.02	−1.06, 5.09	0.19
	Salbutamol	−0.56	−4.84, 3.72	0.80
	Ozone	−2.53	−6.82, 1.75	0.24
	Salbutamol × Ozone	0.79	−5.22, 6.80	0.80
	*R* ^2^ Marginal	0.03		
	*R* ^2^ Conditional	0.00		

** Indicates *p* values less than 0.01.

*** Indicates *p* values less than 0.001.

**TABLE 4:** Model predicted values.

	O_3_ + PLA	O_3_ + SAL	RA + PLA	RA + SAL
% Change in FVC	−1.37 (−2.28, −0.47)	0.77 (−0.14, 1.67)*	−1.44 (−2.35, −0.54)	0.39 (−0.51, 1.29)*
% Change in FEV_1_	−1.38 (−3.12, 0.35)	6.98 (5.25, 8.71)*	−0.35 (−2.08, 1.39)	5.96 (4.23, 7.69)*
Change in FEV_1_/FVC	−0.051 (−1.27, 1.17)	4.56 (3.33, 5.78)*	0.82 (−0.40, 2.04)	4.23 (3.00, 5.45)*
% Change in FEF_25‐75_	−1.00 (−6.70, 4.69)	22.4 (16.7, 28.1)*	3.88 (−1.81, 9.58)	20.2 (14.5, 25.9)*
Change in FeNO (ppb)	−0.52 (−3.50, 2.47)	−0.29 (−3.27, 2.70)	2.02 (−1.07, 5.10)	1.46 (−1.53, 4.45)

*Note*: Values are reported as estimated marginal means and 95% confidence intervals. Values are reported as estimated marginal means and 95% confidence intervals (low, high).

* Indicates *p*‐values less than 0.05 for pairwise post‐hoc comparison. *p*‐values less than 0.05 were found for comparisons between salbutamol versus placebo in room air condition and between the salbutamol versus placebo in Ozone condition.

### 3.2 Overall results

### 3.3 Spirometry

**FIGURE 2:**
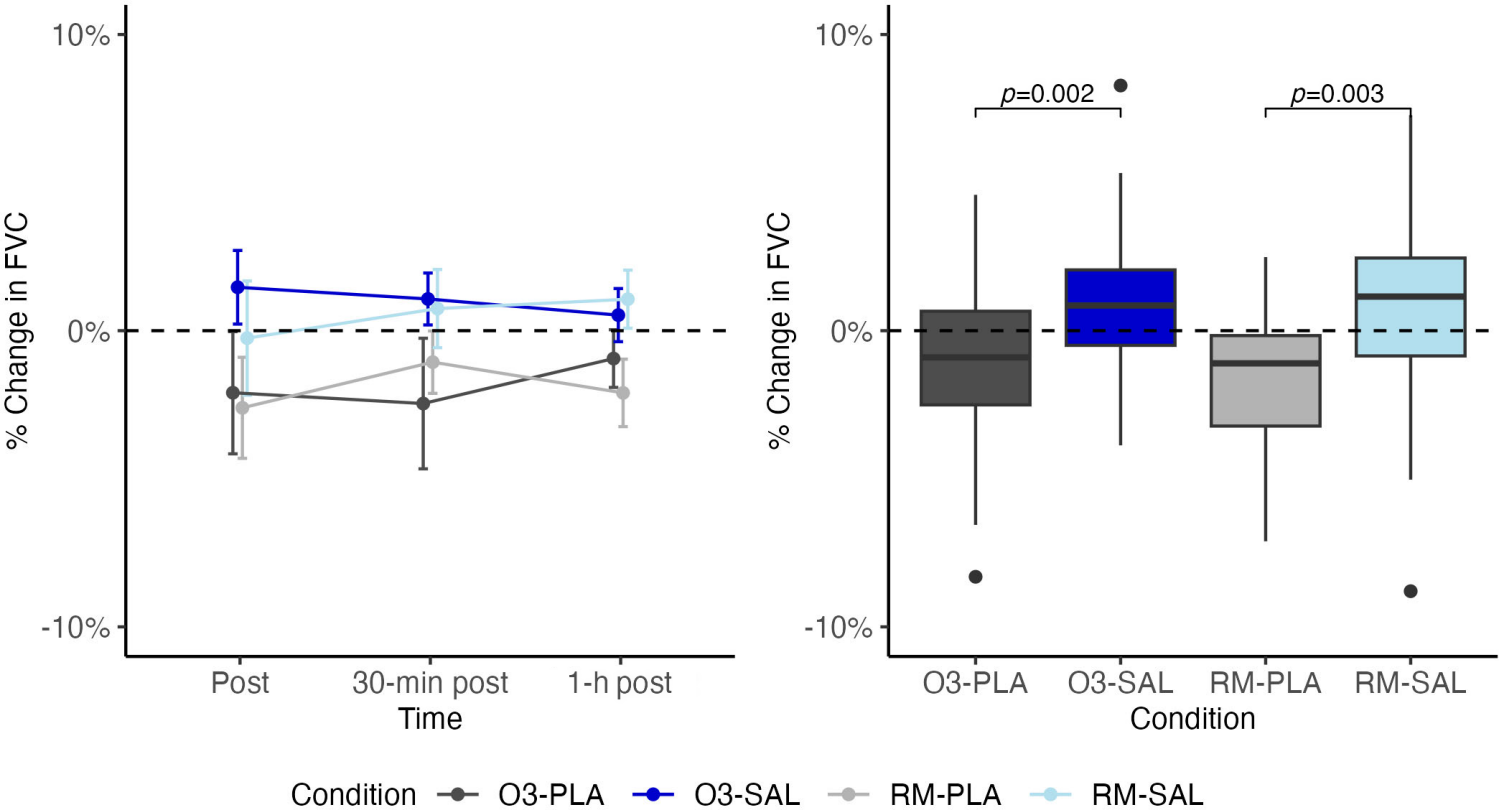
% Change in FVC from baseline line and box plot.

FVC trajectories over the three post‐exercise time periods and boxplots that show the median and interquartile range across all three time points are displayed in Figure 2.

**FIGURE 3:**
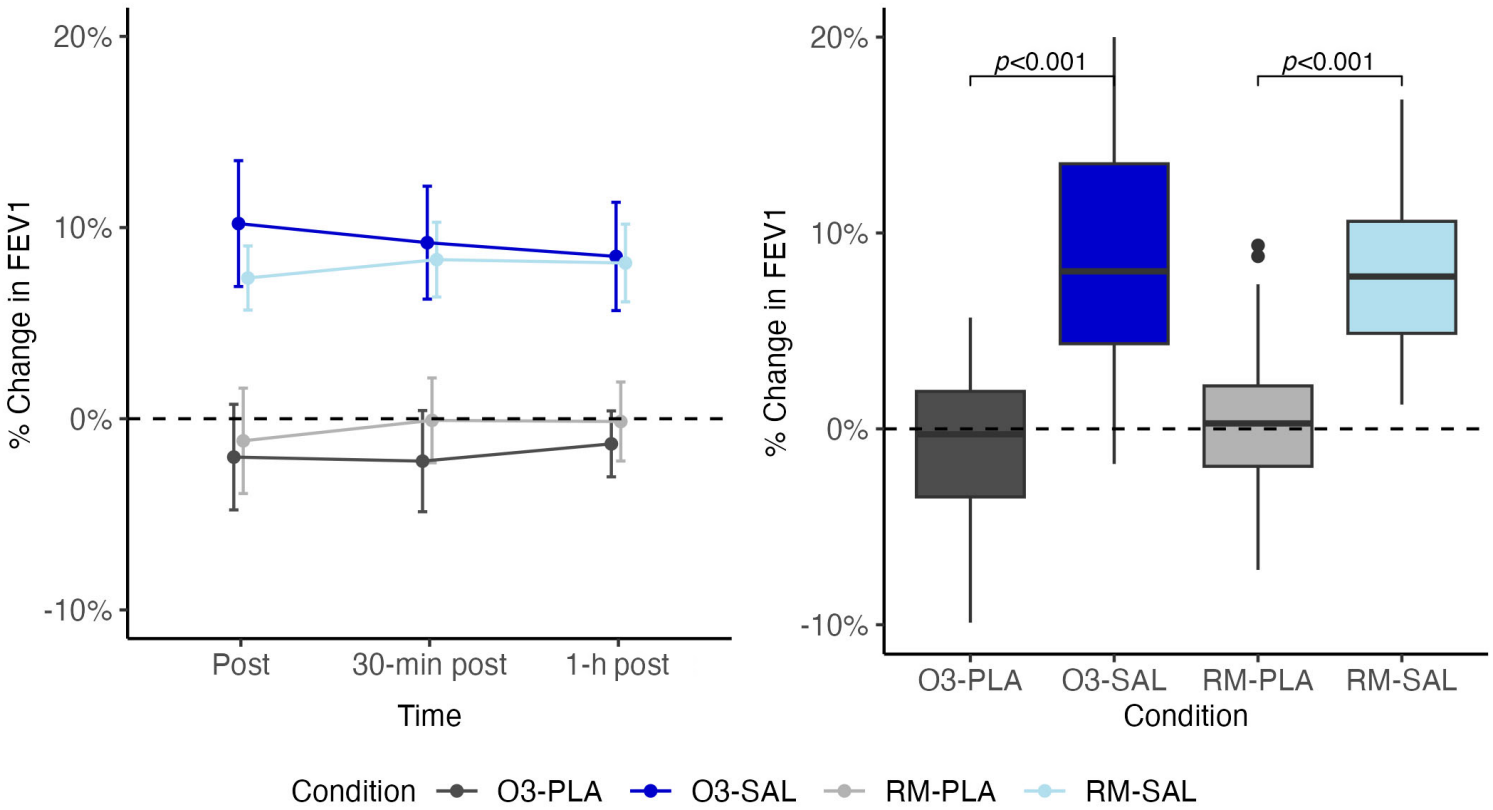
% Change in FEV_1_ from baseline line and box plot.

There was a significant effect of salbutamol on the change in FVC after exercise. We did not find a significant effect of ozone on FVC or a significant interaction effect between salbutamol and ozone. FEV_1_ trajectories and boxplots demonstrate a significant effect of salbutamol when compared to placebo (Figure 3).

**FIGURE 4:**
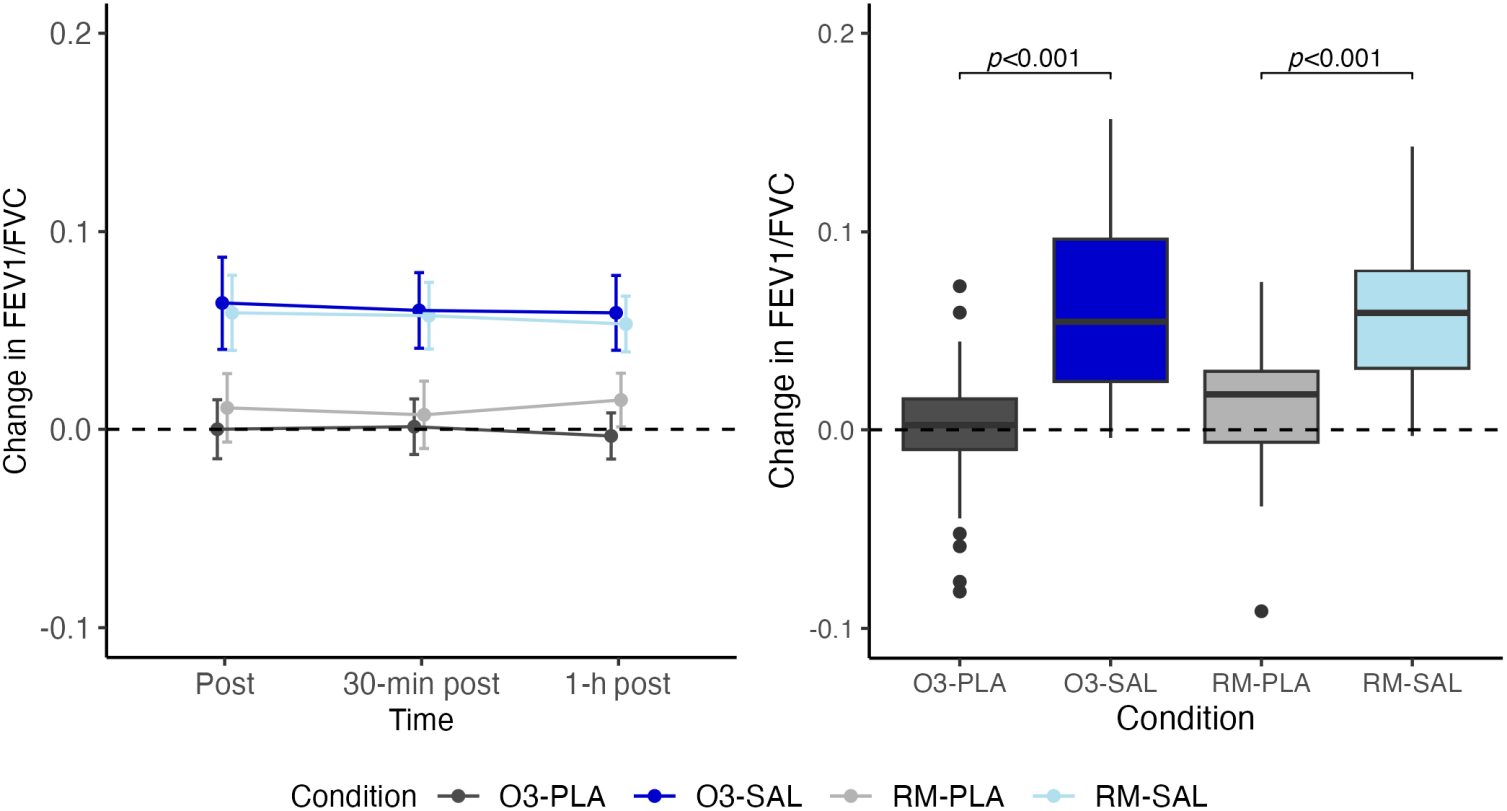
Change in FEV_1_/FVC from baseline line and box plot.

There was no significant effect of ozone when compared to room air or significant interaction between ozone and salbutamol. Similar to FEV_1_, we found a significant effect of salbutamol on FEV_1_/FVC, but no effect of ozone and no interaction between ozone and salbutamol (Figure 4).

**FIGURE 5:**
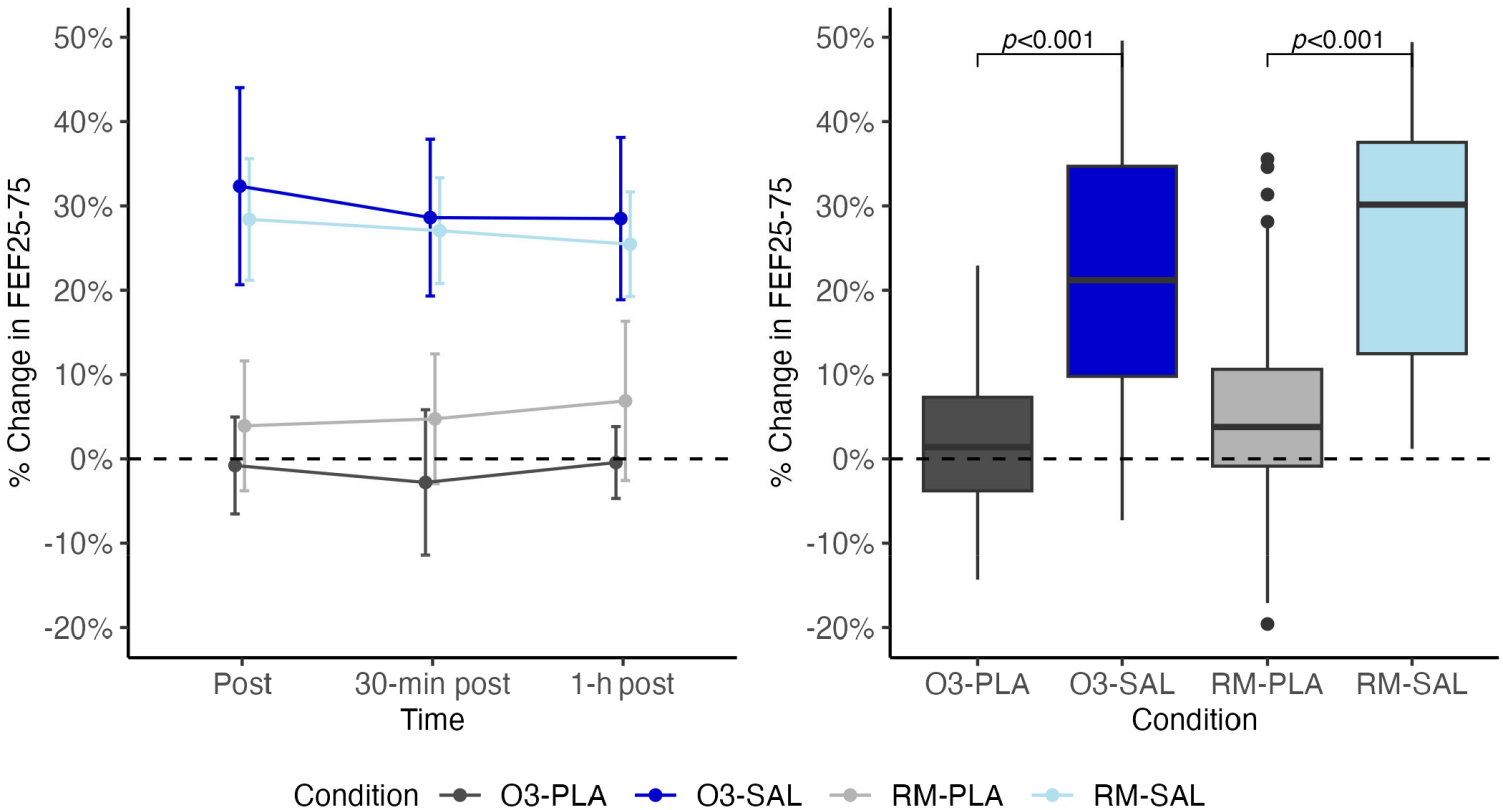
% Change in FEF_25‐75_ from baseline line and box plot.

Regarding FEF_25‐75_, we found a significant effect of salbutamol when compared to placebo, with no effect of ozone or interaction between ozone and salbutamol as shown in Figure 5.

FEF_25‐75_ had a similar trend to FEV_1_, but there were larger changes after exercise and more variability.

### 3.4 FeNO

**FIGURE 6:**
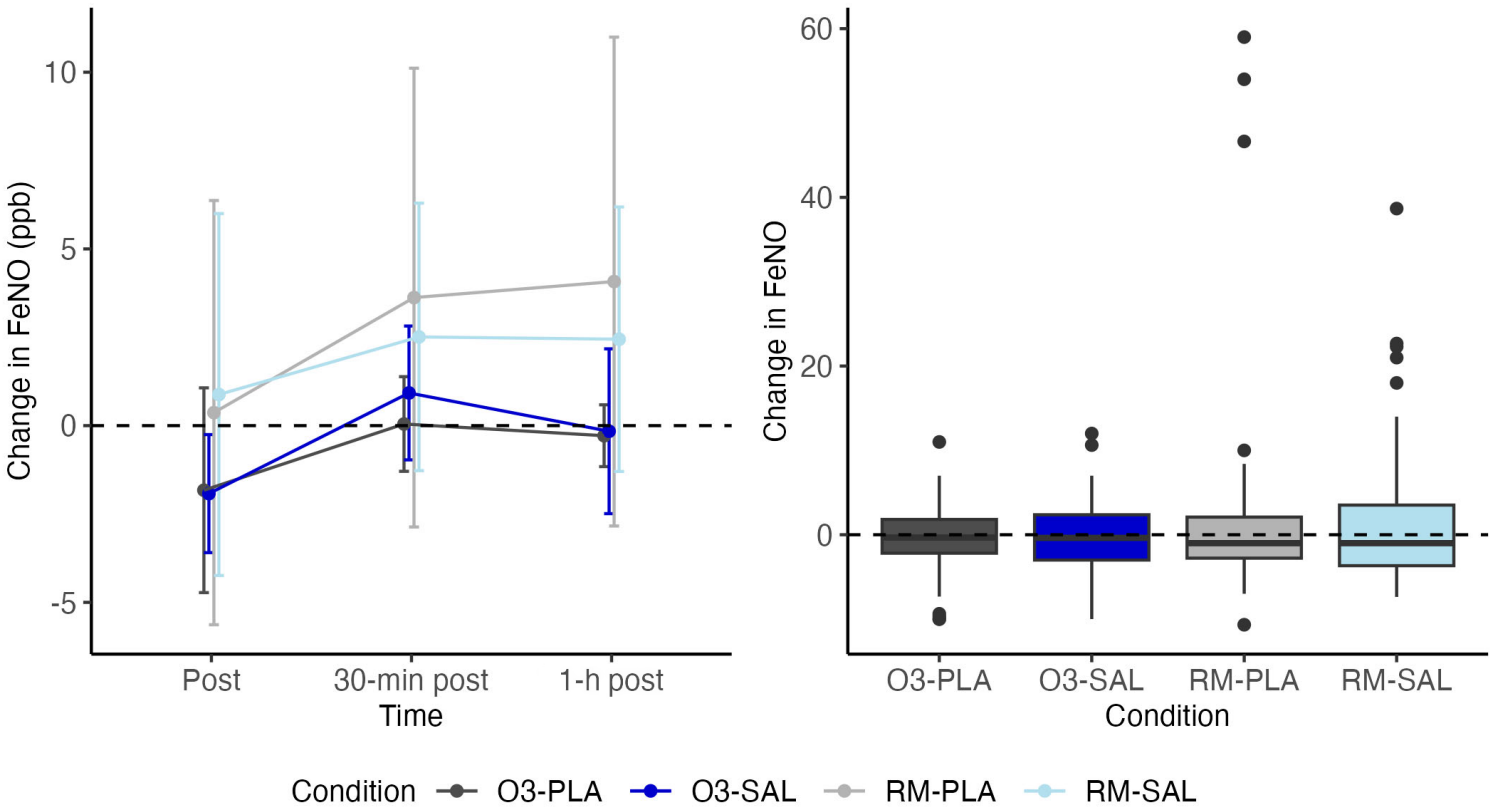
Change in FeNO from baseline line and box plot.

FeNO trajectories and boxplots are presented in Figure 6.

We found no effect of salbutamol, ozone, or an interaction between ozone and salbutamol.

### 3.5 Symptoms

**FIGURE 7:**
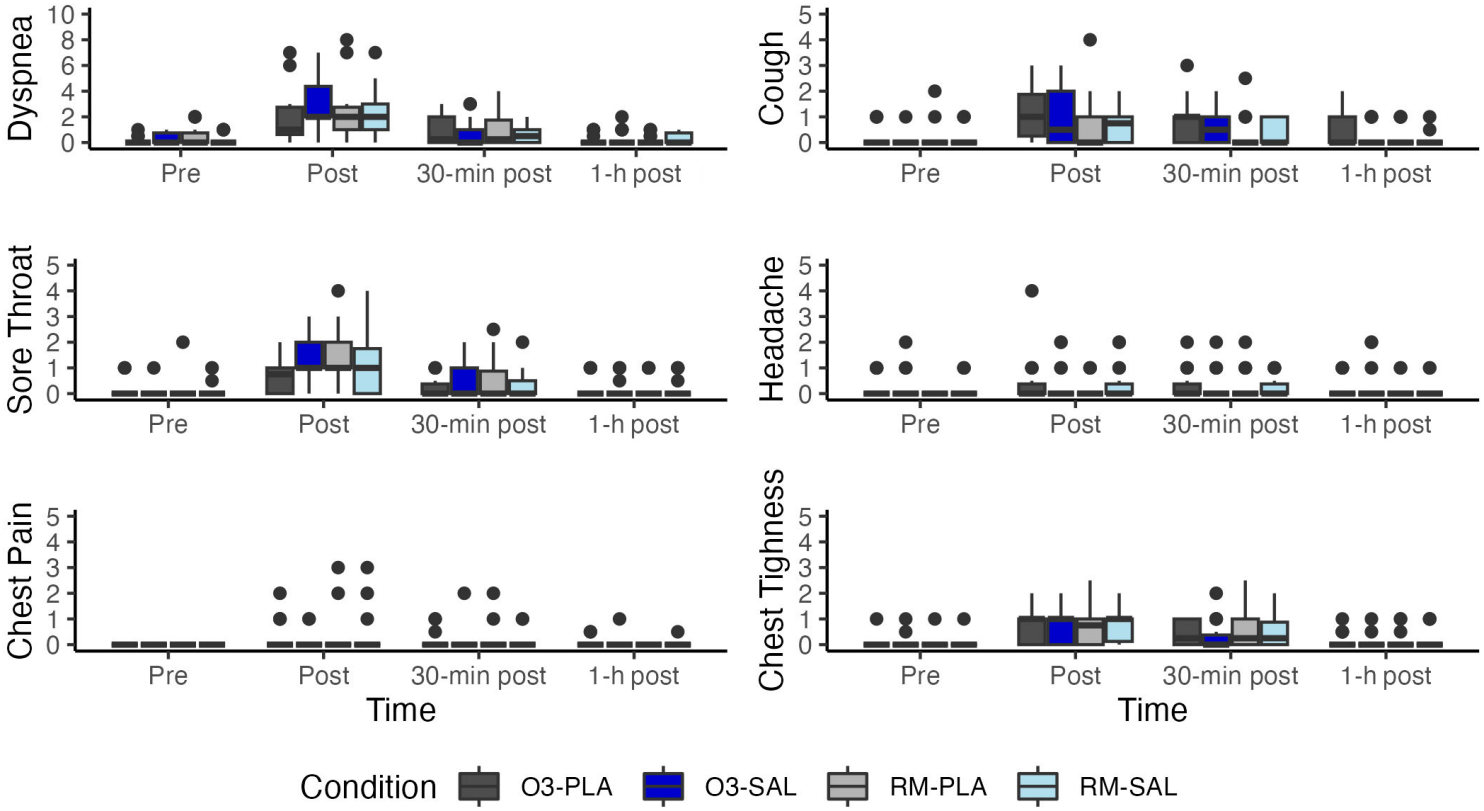
Box plots of symptom scores before, post, 30 and 60 min after exercise.

Boxplots summarizing the symptom scores before, immediately post, 30 min post, and 60 min post are shown in Figure 7.

Symptom scores were low for the majority of the participants with dyspnea generally being less than five out of 10 and the other scores being less than 2.5 out of 10. There were no meaningful differences in symptom severity or frequency between conditions.

## 4 DISCUSSION

We found that salbutamol had a positive effect on pulmonary function and did not significantly effect FeNO regardless of air quality condition. Salbutamol had a statistically significant positive effect on FVC, FEV_1_, FEV_1_/FVC, and FEF_25‐75_ in both the room air and ozone conditions, with the largest effects being seen in FEV_1_ and FEF_25‐75_. Salbutamol did not have a significant effect on FeNO compared to placebo. Ozone did not have a significant effect on any of the outcome variables when compared to room air, in either salbutamol or placebo conditions. We found that participants experienced comparable symptom severity across all conditions. This supported our hypothesis that salbutamol, compared to placebo medication, would improve pulmonary function in ozone; however, contrary to our hypothesis, salbutamol did not exacerbate ozone‐induced airway inflammation. These results provide evidence for the safety of people with EIB to continue to use salbutamol as prescribed even in the presence of high levels of ozone air pollution.

### 4.1 Spirometry

Previously, the effect of salbutamol has only been examined in healthy individuals. For example, Gong et al. showed that salbutamol did not prevent ozone‐related decreases in pulmonary function in healthy competitive cyclists (Gong et al., 2010). By contrast, the asthmatic participants in the present study did demonstrate improved pulmonary function with salbutamol use. Estimated marginal means predicted FEV_1_ to be 6% larger and FEF_25‐75_ to be 20% larger after exercise with salbutamol use compared to placebo. This magnitude of response is comparable to the effect under unpolluted conditions in individuals with EIB after exercise (Aggarwal et al., 2018; Shin et al., 2006). The difference between our findings and those of Gong et al. likely relate to the higher ozone concentration used (210 ppb vs. 170 ppb), and the higher minute ventilations of elite athletes, exercising at higher intensity than in the current study.

We found no significant interactions between salbutamol and ozone, indicating that salbutamol improved pulmonary function to a similar magnitude in both ozone and room air. Small differences in spirometry were observed between room air and ozone in the placebo conditions, with estimated marginal means predicting changes in FEV_1_ and FEF_25‐75_ to be 1.04% and 4.89% higher, respectively, in room air compared to ozone. However, these differences were not significant and were also not observed in the salbutamol conditions. Our findings of FEV_1_ and FEF_25‐75_ being lower in ozone without medication is similar to other ozone exposure studies that found larger decreases in FEV_1_ and FEF_25‐75_ after exposing people with asthma to ozone compared to filtered air. (Kreit et al., 1989) We found a smaller difference than Kreit et al. potentially related to a lower dose of ozone a shorter period of exposure. Additionally, our study employed a higher exercise intensity compared to the intermittent low intensity protocols used in the past. (Kim et al., 2012; Kreit et al., 1989; Weinmann et al., 1995) We interpret our observations to mean that exercise‐induced bronchodilation likely blunted the pulmonary effects of ozone in the placebo conditions.

### 4.2 FeNO

There was no effect of ozone or salbutamol on FeNO post‐exercise confirming the uncertain relationship between FeNO and ozone in the literature. For example, correlations between FeNO and ozone air pollution are reported in some studies (Chen et al., 2020; Niu et al., 2018; Samoli et al., 2017), but not others (Barath et al., 2013; Li et al., 2018). A potential explanation for this discrepancy is that FeNO is specific to the type two inflammatory cascade (Escamilla‐Gil et al., 2022). Thus, a cohort with a predominantly type two inflammatory response may have elevated FeNO in response to ozone while those without this phenotype may not. Our lack of a FeNO response to ozone contradicts the rodent work showing that bronchodilators exacerbated ozone‐related lung inflammation (Henriquez et al., 2019). This could relate to the much higher ozone concentration used with the rodents, or the means of measuring inflammation. FeNO may not be as sensitive to ozone‐induced lung inflammation as the bronchoalveolar lavage used by Henriquez et al., further contributing to the discrepancy. These findings also indicate that FeNO is not a useful tool to clinically assess ozone response in people with asthma. Perhaps albumin, TNF‐α, and Il‐6 from bronchoalveolar lavage, as used by Henriquez et al. would be more sensitive.

### 4.3 Symptoms

We observed no significant differences between the four exposures for respiratory symptoms or other perceptual responses. This finding indicates that the exercise challenge in 170 ppb ozone was insufficient in this population. One could argue that greater concentrations of ozone or more prolonged exercise tasks might have precipitated more symptoms. However, the exercise task was chosen to be representative of a typical workout and the ozone concentration was at the extreme limit of what could actually be experienced under ambient conditions.

### 4.4 Limitations

Despite the strength of the double‐blind placebo‐controlled crossover design, several important limitations exist. First and foremost is the lack of observed pollution effect. However, the findings from the present study are consistent with our previous work were the absence of a pollution effect is seen with acute air pollution exposure and high intensity exercise (Giles et al., 2018), even in asthmatic populations (Koch et al., 2021). In these studies, it appears that the profound physiological responses that accompany whole‐body exercise mask the comparably small acute pollution effect except in studies that have very high pollution levels (Gong et al., 2010), or in elite athletes capable of sustaining very high minute ventilations (Gong et al., 2010; Harris et al., 2024). In designing our experiment, we purposely chose exercise intensities and pollution dose to be representative of a typical moderate exercise bout under severe ozone conditions. Although a higher ozone concentration could have precipitated more of an ozone effect, its generalisability becomes limited.

In choosing participants with EIB our study was also hampered by the limitations of the EVH test we used to screen for EIB. All of our participants had a positive EVH test however, reproducibility of a positive EVH test is poor for milder cases of EIB (Hull et al., 2016).

FeNO, as a measure of inflammation, may not have been sensitive to ozone‐related airway irritation. Previous work used bronchoalveolar lavage fluid to assess inflammation (Weinmann et al., 1995). Although potentially more specific to ozone toxicity, bronchoalveolar lavage would have been significantly more invasive, increasing participant discomfort and making recruiting and data collection more difficult. Another confounding factor is that FeNO can remain elevated for up to an hour after spirometry measurement (Silkoff et al., 2012). This may have artificially inflated our FeNO measures at the 30‐min and 1‐h post‐exercise timepoints. We also only measured FeNO for up to 1 h after exercise and therefore may have missed a potential delayed effect of O_3_ on FeNO. Lastly, our study was conducted in a laboratory setting with a single pollutant exposure. Further work could focus on studying a more complex mixture of air pollutants to better generalize the findings to outdoor exposure.

## 5 CONCLUSION

We found that salbutamol use prior to exercise in high ozone‐containing air improved pulmonary function and that this improvement was comparable to the improvement seen in room air. However, the effect of salbutamol on eosinophilic airway inflammation, as indicated by FeNO, in participants exposed to ozone was minimal. Since salbutamol did not exacerbate the effects of ozone pollution, this study provides evidence for the safety of salbutamol in this population prior to exercising in ozone air pollution in the acute setting.

## FUNDING INFORMATION

This project received funding from the Canadian Network for Research and Innovation in Machining Technology, Natural Sciences and Engineering Research Council of Canada (Grant reference number: RGPIN‐2016‐03754 RTI‐2021‐00091).

## CONFLICT OF INTEREST STATEMENT

The authors declare no conflicts of interest.

## ETHICS STATEMENT

This project received approval from the University of British Columbia Clinical Research Ethics Board (H21‐01080) and conducted according to the declaration of Helsinki. All participants provided written consent after reviewing a consent form.

## Data Availability

Data from this project is available upon reasonable request.
